# Risks and mitigations of substance use disorders with dental procedures-related opioid prescriptions: A systematic review

**DOI:** 10.1016/j.dadr.2026.100467

**Published:** 2026-08-09

**Authors:** Ruben Mikaelyan, Alex Alvarado, Carol Kunzel, Sean X. Luo

**Affiliations:** a Columbia University College of Dental Medicine, New York, NY 10032, USA; b Department of Psychiatry, Columbia University, New York, NY 10032, USA

**Keywords:** Opioid use disorder, Opioid prescriptions, Substance use disorder, Prescription drug monitoring program, Oral surgery prescriptions, Medicaid opioid, Prescriptions

## Abstract

Dental opioid prescriptions remain one of the most common initial exposures to opioids worldwide. Although opioid prescribing in dentistry has declined, even limited exposure to opioids may be a significant risk factor for developing Opioid Use Disorder (OUD), particularly in younger populations. This review evaluates the existing literature on dental opioid prescriptions and the development of OUD, identifies procedural and patient-related risk factors, and assesses the effectiveness of existing intervention strategies. Following the PRISMA guidelines, a comprehensive literature search was conducted across MEDLINE, Embase, PsycINFO, Web of Science, and CENTRAL from January 2011 to July 2026. Eligible studies examined opioid prescribing in dental alveolar surgeries, related OUD/SUD outcomes, and preventative interventions. From an initial 1008 identified records, 12 studies met the final inclusion criteria for qualitative synthesis. Observational studies showed that factors significantly associated with persistent opioid use or adverse outcomes included higher prescribed opioid doses, prior substance use disorder, public or Medicaid insurance, younger age, female sex, and complex chronic medical conditions. Notably, patients undergoing low-pain dental procedures who received opioids demonstrated the highest relative risk for short-term persistent use. Five studies evaluated interventions, finding that short behavioral counseling and opioid-sparing pharmacologic strategies, specifically liposomal bupivacaine and preemptive intravenous paracetamol, effectively reduced postoperative opioid consumption. However, none of the included intervention studies had sufficient follow-up to evaluate the direct prevention of clinical OUD. Future research should prioritize prospective longitudinal studies and randomized controlled trials to evaluate interventions that safely reduce opioid exposure and definitively prevent OUD following dental procedures.

## Highlights

• Dental opioid prescribing contributes to early opioid exposure worldwide.
• Youth and Medicaid patients receive dental opioids most often.
• Dental opioid exposure shows a dose-dependent OUD risk.
• Extractions account for most dental opioid prescriptions.
• Dental prescribing remains a key pathway into opioid misuse.

## 1 Introduction

Opioid analgesics have long served as a cornerstone of acute pain management in outpatient medicine and dentistry globally. In pediatrics and young adults, prescription rates for opioids increased by 35% from 2005 to 2007–2008–2010, then decreased by 14% from 2008 to 2010–2011–2013, and decreased again by 22% from 2011 to 2013–2014–2016 (Pielech et al., 2024). In a 2021 survey of U.S. dentists, 48% reported regularly prescribing opioid medications (Heron et al., 2022). Among adolescents and young adults, tooth extractions accounted for 89% of dental opioid prescriptions (Chua et al., 2026). There is ongoing clinical controversy about whether and to what extent non-opioid analgesic regimens should be prioritized in the management of acute dental pain. A recent large pragmatic randomized clinical trial (Feldman et al., 2025) showed that non-opioid analgesics following impacted third-molar extractions provided effective and potentially superior pain control compared with standard opioid treatment. Initial exposure, even when clinically indicated, may precipitate opioid use disorder (OUD) and other substance use disorders (SUDs) (Campbell et al., 2021), and younger age may be a covariate, but these aspects rely more on clinical experience than a firm empirical basis (Chua et al., 2021b, Schroeder et al., 2019). While there has been an overall decrease in prescribing opioids after dental procedures (Chua et al., 2026), there is evidence-based consensus that the current prescription levels are still too high, as opioids have not been shown to be superior to non-opioid analgesics for postoperative tooth extraction pain management (Feldman et al., 2025).

Dental procedures are among the most common indications for opioid prescribing and therefore represent an important source of initial opioid exposure (Schroeder et al., 2019). Clinically, practitioners often consider younger age, procedure-related pain severity, and ability to adhere to prescription as predictors of developing OUD/SUD, but it is unclear if these ideas are consistent across different studies. No existing systematic review has jointly examined treatment and patient factors and the development of diagnosable OUD in dental settings. Existing systematic reviews focus on the distinctions between different types of pain management options (i.e., opioid vs. non-opioid) (Moore et al., 2018) or the potential liability in prescribing opioids in light of possible addictive potential (Dana et al., 2018). Furthermore, the development of other associated substance use disorders (SUDs) outside of opioids has not been systematically examined. To capture evidence generated during the contemporary era of opioid prescribing, this review specifically examines literature from 2011 onwards. This starting point was intentionally selected to align with the U.S. Food and Drug Administration's (FDA) implementation of the Risk Evaluation and Mitigation Strategy (REMS) for extended-release opioids, which effectively marks the beginning of modern clinical guidance and federal intervention regarding opioid misuse.

The present study aims to: (1) systematically quantify the literature to examine the existing evidence regarding associations between dental opioid exposure and subsequent OUD or SUD outcomes, (2) identify patient-, procedure-, and prescriber-related characteristics associated with heightened risk of developing OUD, and (3) evaluate the effectiveness of current interventions designed to reduce opioid exposure or misuse in dental contexts to prevent OUD. This review seeks to inform the development of targeted educational, clinical decision support, and policy interventions to advance safe and equitable pain management in dentistry and oral surgery.

## 2 Methods

### 2.1 Protocol

A research protocol was developed based on PRISMA guidelines and registered with the Prospective Register of Systematic Reviews (PROSPERO, CRD42024536896).

### 2.2 Eligibility criteria

Studies were included if they met any of the following criteria: (1) reported opioid prescribing practices in dental or oral surgical settings, (2) examined the development of OUD or SUD in relation to patient-specific, procedure-specific, or prescriber-specific factors, (3) were specific to dental alveolar surgery or treatment as opposed to orthognathic surgery, which is substantially more invasive and has distinct postoperative analgesic requirements.

The exclusion criteria included: (1) studies that did not specify the setting as dental or did not isolate the dental context from general medical practices, (2) studies not exclusive to opioids, (3) commentaries, editorials, reviews, and non-empirical studies, (4) studies that were not exclusively focused on dental alveolar surgeries, (5) studies reported on prescription patterns (eg, dosage, duration, or frequency) or qualitative data on prescribers’ decision-making or rationale, (6) studies that evaluated knowledge, attitudes, or adherence of dental professionals to opioid prescribing guideline, (7) studies assessed prescriber behavior using audits, chart reviews, or self-reported practice surveys.

### 2.3 Information sources & search

Two independent reviewers screened titles and abstracts against the inclusion and exclusion criteria. MEDLINE, Embase, PsycINFO, Web of Science, and CENTRAL were searched (January 2011 – July 2026). The search strategy included three core domains: (1) dental settings and procedures (e.g., dentistry, tooth extraction, oral surgery), (2) opioid exposures (e.g., opioids, hydrocodone, oxycodone), and (3) adverse outcomes (e.g., opioid use disorder, substance use disorder, addiction, overdose). The complete, reproducible search strategy for all databases is available in Appendix A.

This timeframe was selected to capture evidence generated during the contemporary era of opioid prescribing following the introduction of national opioid risk-mitigation initiatives, including the U.S. Food and Drug Administration’s Risk Evaluation and Mitigation Strategy (REMS) for opioid analgesics.

### 2.4 Selection of sources of evidence

**Fig. 1:**
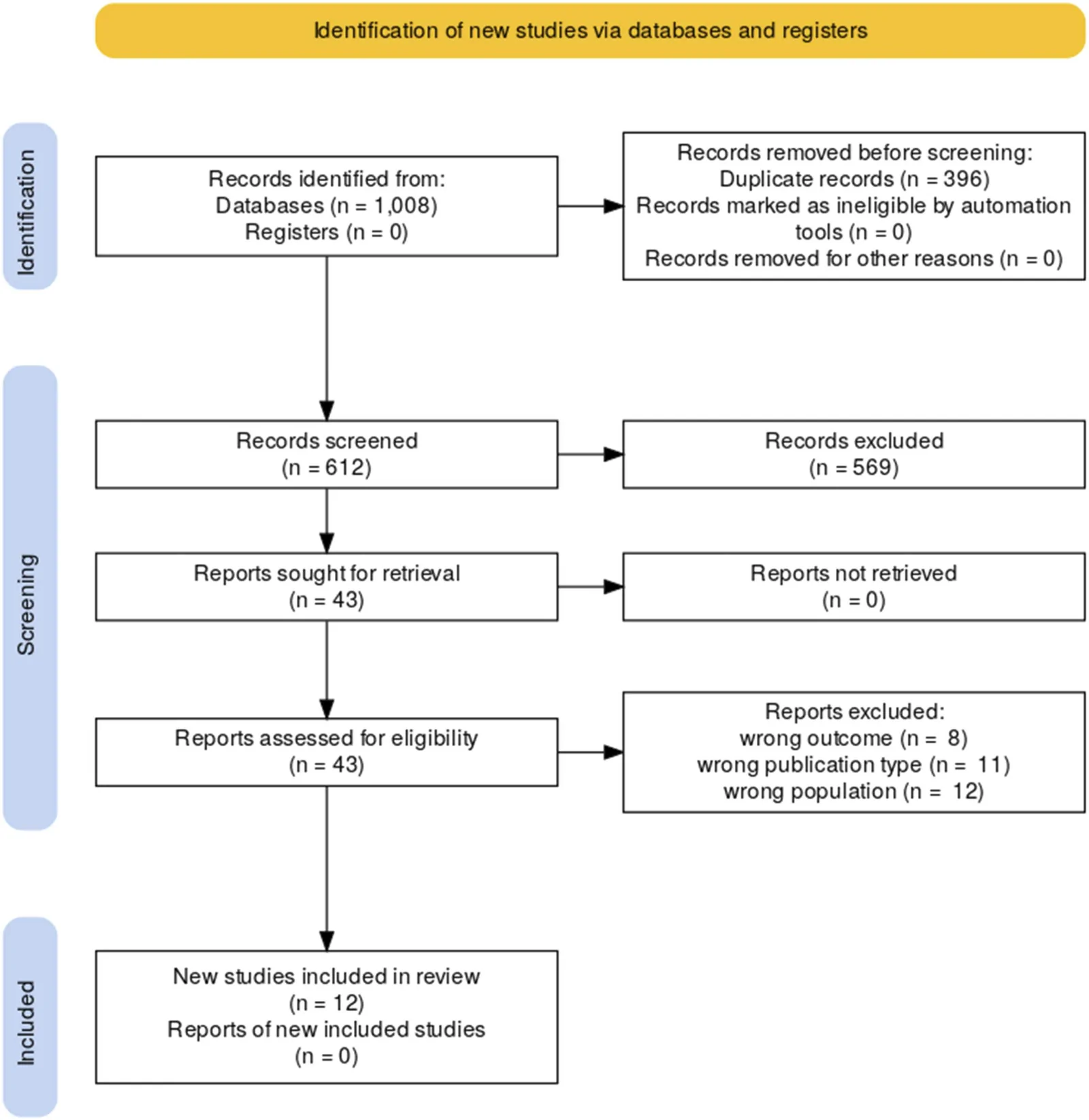
PRISMA Diagram: Risks and Mitigations of Substance Use Disorders with Dental Procedures.

Search results were imported into Rayyan™ and de-duplicated. Two independent reviewers (SL and RM) screened titles and abstracts using the inclusion and exclusion criteria. The reviewers met to reach a consensus on any disagreements. Full texts were obtained for articles that progressed past the abstract screening and were reviewed again by two independent reviewers using the eligibility criteria. Any disagreements at the full-text stage were discussed until consensus was reached. Ultimately, 12 articles were included. For each included study, data were extracted regarding study design, setting, sample characteristics, opioid-prescribing metrics, patient-specific risk factors, and OUD metrics. The studies were then grouped into thematic categories for qualitative analysis. A full PRISMA flow diagram (Fig. 1) depicting the identification, screening, eligibility, and inclusion steps accompanies the manuscript.

### 2.5 Risk of bias assessment

A standardized, seven-domain assessment framework was used to compare studies across a heterogeneous mix of designs using the Cochrane Risk of Bias 2 (RoB 2,) and Risk of Bias in Non-randomized Studies - of Interventions (ROBINS-I). These frameworks evaluated risk across the following domains: Confounding, Selection Bias, Exposure Classification, Missing Data, Outcome Measurement, and Overall Risk. For observational cohorts and case-control studies, the risk assessment was guided by the principles of the Newcastle-Ottawa Scale (NOS), focusing on cohort selection and multivariable adjustment for confounders. Additionally, the studies were assessed for bias with respect to urban versus rural geography and academic versus clinical settings to examine whether these study characteristics might be overrepresented in the literature. The overall risk of bias for each study was synthesized based on the highest level of bias identified within its critical domains.

## 3 Findings

### 3.1 Characteristics of the studies meeting inclusion criteria

A total of 1008 records were identified through database searching and imported into Rayyan™, of which 396 duplicates were removed. Following title and abstract screening, 43 articles underwent full-text review for eligibility. Twelve studies met the inclusion criteria and were included in the qualitative synthesis (Fig. 1). The included studies comprised predominantly retrospective observational cohort studies, with additional prospective interventional and randomized clinical studies evaluating strategies to reduce postoperative opioid exposure. Seven studies examined factors associated with persistent opioid use or opioid-related adverse outcomes following dental opioid prescribing, while five studies evaluated interventions intended to reduce postoperative opioid exposure. Detailed characteristics of the included studies are summarized in Table 2. Risk of bias assessment (Table 1) demonstrated an overall moderate risk of bias across the included studies. Most observational studies exhibited low risk of selection bias because dental procedures represented clearly defined exposure events. However, moderate risk of bias was frequently identified due to missing data, residual confounding, and study populations selected from specific healthcare systems or administrative databases, which may limit generalizability. The major findings of the included studies are presented in the following sections.

**Table 1:** Assessment of Quality and Risk of Bias of the studies included. In addition to the methodological factors in bias we also assessed for preponderance of study geography (Urban/Rural/Mixed) or study setting (academic, community practice or mixed sampling).

Study	Confounding	Selection Bias	Exposure Classification	Missing Data	Outcome Measurement	Study Geography	Study Setting	Overall Risk
Campbell et al. (2021)	Moderate	Low-Moderate	Low	Low-Moderate	Low	Mixed	Mixed	Moderate
Chua et al. (2021)	High	Low	Low	Moderate	Low	Mixed	Mixed	Moderate
Derefinko et al. (2020)	Low	Low	Low	Moderate	Low	Unknown	Unknown	Low
Harbaugh et al. (2018)	Moderate	Low	Low	Moderate	Low	Mixed	Mixed	Moderate
He et al. (2026)	Low	Low	Low	Low-Moderate	Low-Moderate	Urban	Private Practice	Moderate
Iero et al. (2018)	Low	Low	Low	Low	High	Mixed (Urban)	Private/Community Practice	Moderate-High
Keles et al. (2021)	Low	Low	Low	Low	Low	Urban	Academic	Low
Khouja et al. (2022)	Low	Low	Low	Low	High	Mixed (Urban)	Private/Community Practice	Moderate-High
Khouja et al. (2022b)	Moderate	Low	Low	Moderate	Low	Mixed	Mixed	Moderate
Lieblich et al. (2021)	High	High	Low	Moderate	High	Mixed	Private Practice	High
Liu et al. (2025)	Moderate	Low	Low	High	Low-Moderate	Mixed	Mixed	Moderate
Teoh et al. (2026)	Moderate	Low	Low	Moderate	Low	Mixed	Mixed	Moderate

### 3.2 Factors associated with persistent opioid use and opioid-related adverse outcomes

Seven studies evaluated factors associated with persistent opioid use or opioid-related adverse outcomes following dental opioid prescribing.

Prescription-related factors were identified in two studies. (Campbell et al., 2021) reported that patients initially dispensed more than 90 morphine milligram equivalents (MME) per day had significantly greater odds of persistent opioid use than patients prescribed 20 MME/day or less. (Khouja et al., 2022) likewise found that higher prescribed opioid doses were associated with increased risk of persistent opioid use, although opioid doses exceeding CDC-recommended thresholds were not associated with significantly greater risk of serious opioid-related adverse outcomes.

**Table 2:** Included Study Characteristics.

**Article, year (citation)**	**Study Design**	**Cohort Description**	**Exposure/Intervention**	**Main Findings**	**Effect Size**
Campbell et al. (2021)	Retrospective cohort study	786,125 Ontario residents who were dispensed an initial opioid prescription originating from a dentist between October 2014 and September 2018.	Exposures were characterized on the basis of the average daily dose in milligram morphine equivalents and the duration and formulation (long versus short acting) of the initial prescription.	34,880 individuals (4.4%) out of the 786,125 tested developed persistent opioid use. Individuals who were initially dispensed a daily dose of > 90 MME had significantly greater odds of persistent use compared to those dispensed ≤ 20 MME. 140 individuals experienced an opioid overdose within 90 days of the initial prescription.	AOR for High Daily Dose (>90 MME): AOR = 1.20, 95% Confidence Interval (CI) = 1.07–1.34 Interpretation: Individuals who received an initial opioid prescription with a daily dose of > 90 milligram morphine equivalents (MME) had a 20% higher odds of developing persistent opioid use compared to those who received ≤ 20 MME, after adjusting for other factors. This indicates a moderate increase in risk associated with higher initial doses.
Chua et al. (2021)	Retrospective cohort study	1691,878 dental patients aged 13–64 years who underwent dental procedures between July 1, 2014, and December 31, 2017, using data from the 2014–2018 IBM MarketScan Dental, Commercial, and Multi-State Medicaid research databases. The cohort included both privately insured and publicly insured (Medicaid) patients.	Dispensed opioid prescriptions filled between 7 days before and 3 days after the dental procedure (initial prescription).	Publicly insured patients had a higher risk of developing persistent opioid use compared to privately insured patients after receiving dental opioid prescriptions.	The overall risk of POU was 1.3%. For patients with at least one initial prescription, the risk of POU was 2.1% compared to 1.0% for those without an initial prescription (AME = 1.5 %age points, 95% CI = 1.5–1.6). Publicly insured patients had a higher risk of POU (2.0%) compared to privately insured patients (0.9%). Among publicly insured patients, POU risk was 3.2% with an initial prescription versus 1.3% without (AME = 2.3 %age points, 95% CI = 2.1–2.4). Among privately insured patients, POU risk was 2.0% with an initial prescription versus 0.6% without (AME = 1.3 %age points, 95% CI = 1.2–1.3). The AME of initial prescription was 1.0 %age point higher in publicly insured patients (95% CI = 0.9–1.1).
Derefinko et al. (2020)	Randomized controlled trial	76 patients undergoing dental extraction surgery who were eligible for postoperative opioids.	The intervention group received the opioid misuse prevention program.	The intervention group self-reported lower opioid use (in morphine milligram equivalents) one week post-surgery compared to control, and in a sensitivity analysis (excluding participants with surgical complications), this reduction was statistically significant.	The study found d = 0.42 for the intent-to-treat analysis and d = 0.56 in the sensitivity analysis (excluding those with surgical complications).
Harbaugh et al. (2018)	Retrospective cohort study	Patients aged 13–30 years who underwent wisdom tooth extraction defined by tooth number were examined in the Truven Health MarketScan Commercial and Dental database (July 1, 2009-December 31, 2015).	A filled perioperative opioid prescription, defined as one or more opioid prescriptions filled from 7 days before to 3 days after the wisdom tooth extraction.	A filled perioperative opioid prescription after wisdom tooth extraction was associated with higher odds of persistent opioid use among opioid-naive patients. Consistent with prior work showing no association with major or minor surgery, persistent use was not explained by patient characteristics or tooth impaction alone.	Filling a perioperative opioid prescription was associated with adjusted odds ratio 2.69 (95% CI, 2.10–3.44) for persistent opioid use.
He et al. (2026)	Single-blind randomized controlled trial	93 ASA I–II patients (mean age 17 ± 2.7 years; 51% female) undergoing extraction of four impacted third molars under IV sedation at a single private oral and maxillofacial surgery practice in California between January 2023 and June 2025. Patients were randomized to standard local anesthesia (n = 47) or standard local anesthesia plus liposomal bupivacaine (n = 46).	Liposomal bupivacaine plus standard local anesthesia versus standard local anesthesia alone during third molar extraction.	Liposomal bupivacaine significantly reduced postoperative opioid use and cumulative opioid consumption without increasing postoperative pain compared with standard local anesthesia alone.	Opioid use: 26.1% vs. 53.2% (P = .022); cumulative opioid consumption: 5.7 ± 13.3 vs. 12.4 ± 20.8 MME (P = .015); postoperative pain scores: no significant difference (P = .30).
Iero et al. (2018)	Prospective, randomized, open-label trial	69 adults (34 liposomal bupivacaine; 35 control; mean age 56.8 years; 60.9% male) undergoing full-arch implant surgery at four centers in the United States between August 2015 and March 2016. Patients were ≥ 18 years of age, scheduled for full-arch implant surgery with at least four maxillary and four mandibular extractions, and were opioid-naïve (no daily opioid use for >30 days or opioid use within 3 days before surgery).	Addition of liposomal bupivacaine 266 mg to a standardized opioid-sparing postoperative pain management protocol versus the same opioid-sparing protocol alone. All patients received lidocaine with epinephrine, bupivacaine with epinephrine, ibuprofen, and oxycodone as needed.	Patients receiving liposomal bupivacaine experienced significantly lower cumulative postoperative pain and greater early satisfaction with pain control than controls. Opioid consumption was numerically lower, but reductions in oxycodone use did not reach statistical significance.	At 7 days, cumulative pain was reduced by approximately one-third with liposomal bupivacaine (mandible LS mean difference: −10.38 [95% CI, −18.17 to −2.58]; maxilla: −11.84 [95% CI, −20.13 to −3.55]). Patients were more likely to remain oxycodone-free (RR 2.06 on postoperative day 0), although differences in opioid use were not statistically significant.
Keles et al. (2021)	Prospective randomized controlled clinical trial	70 children aged 3–7 years requiring comprehensive dental rehabilitation under general anesthesia at a university pediatric dentistry clinic in Turkey.	Preemptive intravenous paracetamol (15 mg/kg) administered before dental treatment versus intravenous paracetamol administered at the end of treatment.	Preemptive intravenous paracetamol significantly reduced postoperative pain scores, rescue analgesic requirements, and postoperative opioid consumption compared with administration at the end of treatment.	The preemptive group required significantly fewer rescue analgesics (11.4% vs. 34.3%; P = .044), had lower total intravenous fentanyl consumption (2.14 ± 6.50 vs. 5.65 ± 8.05 µg/kg; P = .044), and a longer time to first fentanyl administration (61.88 ± 19.32 vs. 51.05 ± 17.70 min; P = .017) than the control group.
Khouja et al. (2022)	Retrospective cohort study	633,387 dental visits among 531,305 commercially insured U.S. adults (≥18 years) with a dental visit and a concurrent opioid prescription between 2011 and 2018.	Dental opioid prescriptions classified as exceeding (>120 MME) or within CDC-recommended morphine milligram equivalent (MME) limits.	Opioid prescriptions from dentists were associated with serious opioid-related adverse outcomes and persistent opioid use. Exceeding the CDC-recommended MME threshold did not significantly increase the risk of serious adverse outcomes but was associated with a higher risk of persistent opioid use. Patients with prior substance use disorders and those undergoing procedures associated with mild pain had the greatest risk of adverse outcomes and persistent opioid use.	Among 633,387 dental visits, 2.6% resulted in a serious opioid-related adverse outcome within 30 days and 16.6% were associated with persistent opioid use. Persistent opioid use was higher for prescriptions exceeding 120 MME (predicted probability 27.4% vs. 25.2%; P < .001), whereas the risk of serious adverse outcomes was similar between prescriptions exceeding and within recommendations (9.0% vs. 9.1%; P = .66)
Khouja et al. (2022b)	Retrospective cohort study	12,121 Pennsylvania Medicaid enrollees who underwent 1345,360 dental procedures categorized by pain levels (low, moderate, high) between 2012 and 2017.	Receiving an initial opioid prescription within 7 days before or 3 days after a dental procedure.	While high-pain dental procedures had a higher probability of initial opioid prescriptions, patients undergoing low-pain procedures who filled an opioid prescription had the highest relative risk of short-term opioid use. This suggests a need to reduce opioid prescribing for low-pain dental procedures.	The study found that the probability of receiving an initial opioid prescription was 2.4% for low-pain procedures, 8.3% for moderate-pain procedures, and 31.8% for high-pain procedures. For short-term opioid use (4–90 days post procedure), the predicted probability was 25.0% for those who filled an initial prescription versus 0.9% for those who did not in the low-pain group, 16.6% versus 1.6% in the moderate-pain group, and 13.5% versus 2.9% in the high-pain group.
Lieblich et al. (2021)	Retrospective cross-sectional study	600 adults (300 liposomal bupivacaine; 300 non-liposomal bupivacaine) aged ≥ 18 years who underwent extraction of at least one partially or fully impacted mandibular third molar at two outpatient oral and maxillofacial surgery centers in the United States.	Use of liposomal bupivacaine (133 mg) following third molar extraction versus no liposomal bupivacaine.	Patients receiving liposomal bupivacaine were prescribed significantly fewer opioids and required fewer opioid prescription refills than patients who did not receive liposomal bupivacaine, without increasing postoperative complications.	Patients receiving liposomal bupivacaine were prescribed 59% fewer opioids than controls (adjusted MMEs: 44.9 vs. 109.5; rate ratio, 0.41 [95% CI, 0.39–0.44]; P < .0001) and had a significantly lower opioid refill rate (3.3% vs. 7.7%; OR, 0.38 [95% CI, 0.16–0.90]; P = .028).
Liu et al. (2025)	Retrospective cohort study	37,269,952 pediatric dental visits among patients younger than 18 years from 2014 to 2019	Prescription of high-risk medications (opioids and/or benzodiazepines) associated with pediatric dental visits.	High-risk medications were prescribed in 0.72% of pediatric dental visits. Among visits involving opioids, 10.1% experienced an opioid-attributable outcome (opioid-related overdose or persistent opioid use), with the greatest risk observed in younger children, females, patients with complex chronic conditions, and Medicaid beneficiaries. The authors concluded that opioid prescribing in pediatric dentistry should be minimized in favor of guideline-recommended nonopioid analgesics.	High-risk medications were prescribed in 0.72% of pediatric dental visits (269,991/37,269,952). Composite adverse outcomes occurred after 4.3% of visits involving high-risk medications. Among opioid-associated visits, 10.1% experienced an opioid-attributable outcome. Significant predictors included age 4–5 years (OR, 1.48; 95% CI, 1.38–1.57), female sex (OR, 1.08; 95% CI, 1.05–1.11), complex chronic conditions (OR, 1.22; 95% CI, 1.14–1.30), and Medicaid insurance (OR, 1.29; 95% CI, 1.25–1.33).
Teoh et al. (2026)	Retrospective cohort study.	97,233 opioid-naïve Australian patients who were dispensed an initial opioid prescription by a dentist between 2015 and 2022, identified from a standardized 10% random sample of the Australian Pharmaceutical Benefits Scheme (PBS) database. Patients were followed for 12 months after the initial prescription.	Initial dental opioid prescription among opioid-naïve patients.	Persistent opioid use developed in a small but clinically significant proportion of opioid-naïve patients following dental opioid prescribing. Patients with POU received substantially more opioid prescriptions during follow-up, were more likely to receive subsequent prescriptions from non-dental providers, and were dispensed higher-potency opioids after the initial prescription.	Of 97,233 opioid-naïve patients, 1.7% (1692) developed persistent opioid use. Patients with POU received opioids at a rate of 4397 vs. 1209 prescriptions per 1000 patient-years (3.6-fold higher), and subsequent opioid prescriptions had a 44.1% higher mean MME (161.4 vs. 112.0 mg; P < .001) than patients without POU.

Multiple patient characteristics were associated with increased risk of developing OUD or persistent opioid use. (Chua et al., 2021) reported that publicly insured patients had a higher risk of persistent opioid use than privately insured patients after receiving dental opioid prescriptions. (Liu et al., 2025) found that 10.1% of pediatric visits involving an opioid prescription resulted in an opioid-attributable outcome, defined as persistent opioid use or opioid-related overdose. Within that cohort, younger age, female sex, Medicaid insurance, and complex chronic medical conditions were associated with increased risk. Khouja et al. additionally identified prior substance use disorder as a risk factor for both persistent opioid use and serious opioid-related adverse outcomes.

Procedure-related findings varied across studies. (Harbaugh et al., 2018) found that opioid-naïve patients who filled a perioperative opioid prescription following third molar extraction had greater odds of persistent opioid use than patients who did not fill an opioid prescription. (Khouja et al., 2022b) reported that although high-pain dental procedures were more likely to receive an initial opioid prescription, patients undergoing low-pain procedures who received opioids experienced the highest relative risk of short-term opioid use. (Teoh et al., 2026) reported that persistent opioid use developed in a small proportion of opioid-naïve patients following dental opioid prescribing. Patients who developed persistent opioid use subsequently received more opioid prescriptions during follow-up, were more likely to receive prescriptions from non-dental providers, and were prescribed higher-potency opioids than patients without persistent opioid use.

In summary, while each individual study identified some risk factors and pointed to a pattern of predictors that might be related to high baseline risk (public insurance use, chronic medical history, and substance use disorder history), these effects were not large or consistent across different studies. The procedure- or dosing-related effects were also inconsistent across different studies, not sufficiently useful as a stratification for a future randomized study.

### 3.3 Interventions to prevent OUD

Five studies evaluated interventions intended to reduce postoperative opioid exposure through behavioral or analgesic approaches.

Derefinko et al. (2020) was a randomized trial that evaluated a behavioral intervention designed to reduce postoperative opioid use. 76 participants received a short 10-min educational counseling intervention, and the treatment group reported lower opioid consumption, measured in morphine milligram equivalents (MME), during the first postoperative week compared with controls. In a sensitivity analysis excluding patients with postoperative surgical complications, the reduction in opioid consumption reached statistical significance. This study was the only bona fide behavioral prevention study for this indication.

Four studies evaluated opioid-sparing pharmacologic analgesic strategies for OUD prevention. Three studies investigated liposomal bupivacaine. He et al. (2026) reported significantly lower postoperative opioid use and cumulative opioid consumption among patients receiving liposomal bupivacaine compared with standard local anesthesia, without increased postoperative pain. Iero et al. (2018) found lower cumulative postoperative pain scores and greater early patient satisfaction with pain control in the liposomal bupivacaine group, while postoperative oxycodone consumption was numerically lower but did not differ significantly between groups. Lieblich et al. (2021) reported that patients receiving liposomal bupivacaine were prescribed fewer opioids and required fewer opioid prescription refills than patients who did not receive liposomal bupivacaine, with no increase in postoperative complications. Keles et al. (2021) evaluated preemptive intravenous paracetamol and found that administration before surgery resulted in lower postoperative pain scores, reduced rescue analgesic requirements, and lower postoperative opioid consumption compared with administration at the completion of treatment.

In summary, the handful of intervention studies suggested that a combination of non-opioid pain management and behavioral interventions might be effective in not only preventing persistent and heavy opioid use but also potentially preventing the emergence of OUD. However, so far the latter outcome has not yet been tested.

## 4 Discussion

This review synthesized the available evidence examining opioid prescribing following dental procedures, factors associated with persistent opioid use and opioid-related adverse outcomes, and interventions designed to reduce postoperative opioid exposure. Several patient-, prescription-, and procedure-related factors were shown to be associated with an increased risk of persistent opioid use, including higher prescribed opioid doses, prior substance use disorder, public insurance, younger age, and undergoing low-pain procedures for which opioids were prescribed. Although relatively few intervention studies specific to dental-alveolar surgery were identified, both behavioral and multimodal analgesic strategies demonstrated potential to reduce postoperative opioid consumption.

The findings of this review reinforce growing evidence that even short-term opioid prescribing in dental settings may have consequences extending beyond the immediate postoperative period. Multiple studies demonstrated an association between higher initial opioid doses and an increased likelihood of persistent opioid use, supporting recommendations to prescribe the lowest effective dose for the shortest duration when opioid therapy is considered necessary. Notably, patients undergoing procedures typically associated with lower postoperative pain experienced a disproportionately greater relative risk of continued opioid use when opioids were prescribed, suggesting that prescribing decisions could be guided by the expected severity of postoperative pain rather than routine practice. Furthermore, several studies identified patient characteristics associated with elevated risk, including prior substance use disorder, Medicaid or public insurance status, younger age, female sex, and complex chronic medical conditions. Recognition of these factors may assist clinicians in identifying patients who could benefit from closer follow-up or consideration of non-opioid analgesic strategies.

Notably, (Teoh et al., 2026) reported that patients who developed persistent opioid use subsequently received more opioid prescriptions, were more likely to obtain prescriptions from non-dental providers, and received higher-potency opioids than patients without persistent opioid use. These findings suggest that dental opioid prescribing may represent the initial point of opioid exposure for some patients while highlighting the importance of coordinated prescribing practices across healthcare disciplines. Dentists could therefore consider postoperative pain management within the broader context of a patient’s healthcare, emphasizing patient education, careful prescribing practices, and communication with other healthcare providers when appropriate.

Some studies also evaluated interventions to reduce opioid exposure. The available evidence suggests that behavioral interventions may reduce postoperative opioid consumption, while opioid-sparing analgesic strategies (e.g, liposomal bupivacaine, preemptive intravenous paracetamol) may decrease opioid requirements. Although these findings are encouraging, the evidence remains limited by the small number of randomized and prospective studies. Furthermore, none of these studies examined the emergence of OUD, which requires a longer duration of follow-up and a larger sample size. Additional high-quality clinical trials comparing non-opioid analgesic protocols with conventional opioid-based pain management can be done to solidify best practices for postoperative dental pain management.

### 4.1 Limitations

This review is limited by the heterogeneity of the included studies, many of which rely on retrospective or administrative data that may not capture nuanced clinical decision-making. The lack of large randomized controlled trials that have a definitive OUD diagnosis restricts the ability to draw causal inferences regarding the efficacy of prevention interventions. Most studies used mixed urban/rural and academic/community settings. Some studies had an academic emphasis. Future studies might consider enrolling rural clinics specifically to identify risks that may differ from those in urban settings. Future research could also consider prioritizing longitudinal and experimental designs to evaluate both patient-centered outcomes and clinician behavior in response to targeted interventions. There is also a need to develop and validate risk prediction models that can be operationalized at the point of care to guide opioid prescribing decisions in dental settings.

## 5 Conclusion

This review found that opioid prescribing following dental procedures is associated with an increased risk of persistent opioid use and opioid-related adverse outcomes in certain patient populations. Higher prescribed opioid doses, prior substance use disorder, younger age, and public or Medicaid insurance were among the factors associated with increased risk, while opioid prescribing following lower-pain dental procedures was also associated with greater subsequent opioid use.

The available evidence also suggests that behavioral interventions and opioid-sparing analgesic strategies, including liposomal bupivacaine and preemptive intravenous paracetamol, may reduce postoperative opioid consumption while maintaining effective pain management.

These findings support the continued use of evidence-based, opioid-sparing approaches to postoperative dental pain management and emphasize the importance of individualized prescribing practices that consider both procedural and patient-related risk factors. Future research could prioritize prospective longitudinal studies and randomized controlled trials using standardized outcome measures to better define the relationship between dental opioid prescribing and long-term opioid-related outcomes and to evaluate interventions that safely reduce opioid exposure following dental procedures.

## CRediT authorship contribution statement

**Carol Kunzel:** Writing – review & editing, Writing – original draft, Visualization, Validation. **Luo Sean:** Writing – review & editing, Writing – original draft, Visualization, Validation, Supervision, Resources, Project administration, Methodology, Investigation, Formal analysis, Data curation, Conceptualization. **Alex Alvarado:** Writing – review & editing, Writing – original draft, Validation, Formal analysis. **Ruben Mikaelyan:** Writing – review & editing, Writing – original draft, Visualization, Validation, Resources, Project administration, Methodology, Investigation, Formal analysis, Data curation, Conceptualization.

## Declaration of Competing Interest

The authors declare that they have no competing financial interests or personal relationships that could be perceived to have influenced the work reported in this paper.
