# Supplementary material for: Risks and mitigations of substance use disorders with dental procedures-related opioid prescriptions: A systematic review
Source: Drug Alcohol Depend Rep. 2026 Aug 9;20:100467. doi: 10.1016/j.dadr.2026.100467 (PMC13521135; doi:10.1016/j.dadr.2026.100467)
Supplement: Supplementary file 1 [file mmc1.docx]

Appendix A:

Database: MEDLINE Date Searched: [Not Specified] Filters Applied: [None Specified] Search String: ((dentist* OR dentistry OR dental).ti. OR ("oral surgery" OR "maxillofacial surgery" OR "tooth extraction*" OR "third-molar extraction*" OR endodontic* OR alveoloplasty OR "dentoalveolar" OR orthognathic*).ti,ab.) AND (opioid* OR opiate* OR hydrocodone OR oxycodone OR codeine OR tramadol).ti,ab. AND ("opioid use disorder*" OR OUD OR "substance use disorder*" OR SUD OR addiction OR misuse OR abuse OR dependenc* OR overdose OR "persistent opioid use" OR "persistent use" OR "subsequent use" OR "chronic use" OR "opioid consumption" OR "morphine milligram equivalent*" OR MME OR "opioid-sparing" OR "opioid sparing").ti,ab. Results Yielded: 181

Database: Embase Date Searched: [Not Specified] Filters Applied: [None Specified] Search String: ('dentistry'/exp OR 'mouth surgery'/exp OR 'tooth extraction'/exp OR dentist*:ti OR dentistry:ti OR dental:ti OR 'oral surgery':ti,ab OR 'maxillofacial surgery':ti,ab OR 'tooth extraction*':ti,ab OR 'third-molar extraction*':ti,ab OR endodontic*:ti,ab OR alveoloplasty:ti,ab OR dentoalveolar:ti,ab OR orthognathic*:ti,ab) AND ('opiate'/exp OR opioid*:ti,ab OR opiate*:ti,ab OR hydrocodone:ti,ab OR oxycodone:ti,ab OR codeine:ti,ab OR tramadol:ti,ab) AND ('substance related disorder'/exp OR 'opiate addiction'/exp OR 'opioid use disorder*':ti,ab OR OUD:ti,ab OR 'substance use disorder*':ti,ab OR SUD:ti,ab OR addiction:ti,ab OR misuse:ti,ab OR abuse:ti,ab OR dependenc*:ti,ab OR overdose:ti,ab OR 'persistent opioid use':ti,ab OR 'persistent use':ti,ab OR 'subsequent use':ti,ab OR 'chronic use':ti,ab OR 'opioid consumption':ti,ab OR 'morphine milligram equivalent*':ti,ab OR MME:ti,ab OR 'opioid-sparing':ti,ab OR 'opioid sparing':ti,ab) Results Yielded: 433

Database: PsycINFO Date Searched: [Not Specified] Filters Applied: [None Specified] Search String: (DE "Dentistry" OR DE "Dental Health" OR TI (dentist* OR dentistry OR dental) OR TI ("oral surgery" OR "maxillofacial surgery" OR "tooth extraction*" OR "third-molar extraction*" OR endodontic* OR alveoloplasty OR dentoalveolar OR orthognathic*) OR AB ("oral surgery" OR "maxillofacial surgery" OR "tooth extraction*" OR "third-molar extraction*" OR endodontic* OR alveoloplasty OR dentoalveolar OR orthognathic*)) AND (DE "Opiates" OR DE "Analgesic Drugs" OR TI (opioid* OR opiate* OR hydrocodone OR oxycodone OR codeine OR tramadol) OR AB (opioid* OR opiate* OR hydrocodone OR oxycodone OR codeine OR tramadol)) AND (DE "Substance Use Disorder" OR DE "Drug Addiction" OR DE "Drug Abuse" OR TI ("opioid use disorder*" OR OUD OR "substance use disorder*" OR SUD OR addiction OR misuse OR abuse OR dependenc* OR overdose OR "persistent opioid use" OR "persistent use" OR "subsequent use" OR "chronic use" OR "opioid consumption" OR "morphine milligram equivalent*" OR MME OR "opioid-sparing" OR "opioid sparing") OR AB ("opioid use disorder*" OR OUD OR "substance use disorder*" OR SUD OR addiction OR misuse OR abuse OR dependenc* OR overdose OR "persistent opioid use" OR "persistent use" OR "subsequent use" OR "chronic use" OR "opioid consumption" OR "morphine milligram equivalent*" OR MME OR "opioid-sparing" OR "opioid sparing")) Results Yielded: 27

Database: Web of Science Date Searched: [Not Specified] Filters Applied: [None Specified] Search String: ((TS=(dentist* OR dentistry OR dental OR "oral surgery" OR "maxillofacial surgery" OR "tooth extraction*" OR "third molar extraction*" OR endodontic* OR alveoloplasty OR dentoalveolar OR orthognathic*)) AND TS=(opioid* OR opiate* OR hydrocodone OR oxycodone OR codeine OR tramadol)) AND TS=("opioid use disorder*" OR OUD OR "substance use disorder*" OR SUD OR addiction OR misuse OR abuse OR dependenc* OR overdose OR "persistent opioid use" OR "persistent use" OR "subsequent use" OR "chronic use" OR "opioid consumption" OR "morphine milligram equivalent*" OR MME OR "opioid sparing") Results Yielded: 301

Database: CENTRAL (Cochrane) Date Searched: [Not Specified] Filters Applied: [None Specified] Search String: ((dentist* OR dentistry OR dental):ti OR ("oral surgery" OR "maxillofacial surgery" OR "tooth extraction" OR "tooth extractions" OR "third molar extraction" OR "third molar extractions" OR endodontic* OR alveoloplasty OR dentoalveolar OR orthognathic*):ti,ab,kw) AND (opioid* OR opiate* OR hydrocodone OR oxycodone OR codeine OR tramadol):ti,ab,kw AND ("opioid use disorder" OR "opioid use disorders" OR OUD OR "substance use disorder" OR "substance use disorders" OR SUD OR addiction OR misuse OR abuse OR dependenc* OR overdose OR "persistent opioid use" OR "persistent use" OR "subsequent use" OR "chronic use" OR "opioid consumption" OR "morphine milligram equivalent" OR "morphine milligram equivalents" OR MME OR "opioid sparing"):ti,ab,kw Results Yielded: 67
